# Recent Trends in the Incidence of Clear Cell Adenocarcinoma and Survival Outcomes: A SEER Analysis

**DOI:** 10.3389/fendo.2022.762589

**Published:** 2022-02-24

**Authors:** Yadong Guo, Anil Shrestha, Niraj Maskey, Xiaohui Dong, Zongtai Zheng, Fuhan Yang, Ruiliang Wang, Wenchao Ma, Ji Liu, Cheng Li, Wentao Zhang, Shiyu Mao, Aihong Zhang, Shenghua Liu, Xudong Yao

**Affiliations:** ^1^ Department of Urology, Shanghai Tenth People’s Hospital, Tongji University, Shanghai, China; ^2^ Urologic Cancer Institute, Tongji University School of Medicine, Shanghai, China; ^3^ Department of Urology, National Academy of Medical Sciences, Bir Hospital, Kathmandu, Nepal; ^4^ Department of General Medical, Shanghai Fourth People’s Hospital, Tongji University, Shanghai, China; ^5^ Department of Medical Statistics, Tongji University School of Medicine, Shanghai, China

**Keywords:** clear cell adenocarcinoma, SEER database, incidence, survival, glycogen-rich phenotype

## Abstract

**Background:**

Clear cell adenocarcinoma (CCA) is considered a relatively rare tumor with a glycogen-rich phenotype. The prognosis of CCA patients is unclear. In this study, recent trends in the epidemiological and prognostic factors of CCA were comprehensively investigated.

**Methods:**

Patients with CCA from years 2000 to 2016 were identified from the Surveillance, Epidemiological, and End Results (SEER) database. Relevant population data were used to analyze the rates age-adjusted incidence, age-standardized 3-year and 5-year relative survivals, and overall survival (OS).

**Results:**

The age-adjusted incidence of CCA increased 2.7-fold from the year 2000 (3.3/100,000) to 2016 (8.8/100,000). This increase occurred across all ages, races, stages, and grades. Of all these subgroups, the increase was largest in the grade IV group. The age-standardized 3-year and 5-year relative survivals increased during this study period, rising by 9.1% and 9.5% from 2000 to 2011, respectively. Among all the stages and grades, the relative survival increase was greatest in the grade IV group. According to multivariate analysis of all CCA patients, predictors of OS were: age, gender, year of diagnosis, marital status, race, grade, stage, and primary tumor site (*P* < 0.001). The OS of all CCA patients during the period 2008 to 2016 was significantly higher than that from 2000 to 2007 (*P* < 0.001).

**Conclusions:**

The incidence of CCA and survival of these patients improved over time. In particular, the highest increases were reported for grade IV CCA, which may be due to an earlier diagnosis and improved treatment.

## Background

Clear cell carcinoma (CCA) consists of a series of malignant tumors, likely caused by abnormal deposition of glycogen (1). Glycogen is a branched-chain polysaccharide composed of glucose that is necessary to maintain homeostasis of normal cell metabolism, but can promote tumor growth, especially under adverse conditions (2–4). Studies have shown that hypoxia within the centers of solid tumors leads to an increase in glycogen storage, allowing adaptation to low oxygen levels and lack of nutrients. The possible mechanism involves hypoxia-inducible factor 1α (HIF-1α)-mediated signaling pathways (4, 5). Therefore, the regulation of glycogen synthesis and degradation is crucial for cellular homeostasis. In recent years, more and more studies have emphasized that reprogramming of glycogen metabolism affects the occurrence and progression of malignant tumors; hence, it has become a recognized feature of tumor cells (6, 7). Although a few drugs targeting glycogen metabolism are currently being tested as a component of comprehensive treatment for tumors, they have not yet been approved for clinical application (8, 9).

There is evidence that CCA shows striking similarities in the gene expression profiles of various organs (10). In addition, histological staining of CCA tumors shows “clear cells” with a transparent and oval appearance that are rich in cytoplasmic glycogen. Since CAA has no obvious symptoms, its diagnosis is based on histopathological identification of these characteristics. Previous research also suggests a link between glycogen-rich tumors and tumor aggressiveness, and CCA of the kidney, ovary, and bladder have a poor prognosis and are resistant to treatment (11–14). In addition, although studies have described the effect of CCA on the prognosis of some cancer patients, the epidemiology and survival of CCA patients in the general population have not been well described. Therefore, in this study, we attempted to perform the most complete analysis of the incidence, patient demographics, and prognostic factors of CCA using data from the Surveillance, Epidemiological, and End Results (SEER) program.

## Methods

### Database and Patient Selection

The SEER program is the definitive source of cancer incidence and survival data collected by the National Cancer Institute, covering approximately 34.6% of the US population. Our study used the SEER 18 database and identified all malignancies that were diagnosed as CCA between 1 January 2000 and 31 December 2016: kidney and renal pelvis (KRP); ovary; cervix and corpus uteri; lung and bronchus; urinary bladder; vagina; pancreas; breast; peritoneum, omentum, and mesentery (POM); prostate; and liver. The CD-O-3 histology code was used to identify CCA. This code corresponds to the following clinical/histological diagnoses: 8310/2, 8310/3, and 8313/3. The following information was collected from the database: primary tumor site; ICD-O-3 histology; age at diagnosis; gender; marital status; race; grade; tumor stage in 2000; and sites of metastasis at diagnosis (bone, brain, liver, and lung from years 2010 to 2016). Studies with a lack of survival data or fewer than 50 CCA solid tumors were excluded. The final cohort included 104,206 patients.

### Statistical Analysis

SEER*Stat software (version 8.3.6, National Cancer Institute Surveillance Research Program) was used to calculate the age-adjusted incidence and age-standardized relative survival. Incidence and relative survival were standardized to the 2000 United States general population.

All statistical analyses were performed using IBM SPSS v25.0 software (International Business Machines, Armonk, NY, USA). The data were analyzed to produce figure using Microsoft Excel software. Differences in demographic and clinical characteristics of different primary tumor sites were determined using Pearson’s chi-squared test. Overall survival (OS) was generated using the Kaplan-Meier curves and the difference in survival between groups was assessed using a log-rank test. A multivariate Cox proportional hazards regression model was used to estimate the 3-year, 5-year, and OS for all patients, respectively. Two-tailed *P* values < 0.05 were considered statistically significant.

## Results

### Annual Incidence of CCA

**Figure 1:**
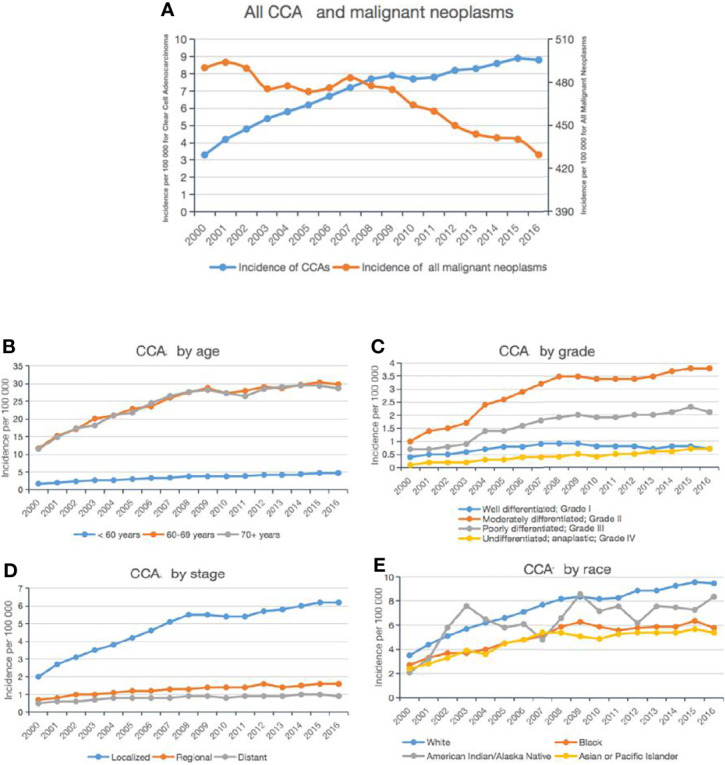
Incidence trends of CCA from 2000 to 2016. Annual age-adjusted incidence of all CCA cases and all malignant neoplasms **(A)**. Annual age-adjusted incidence of CCA by age group **(B)**, stage **(C)**, grade **(D)**, and ethnicity **(E)**.

To assess the recent trends in CCA incidence, we identified all CCA cases from 2000 to 2016 in the SEER database. As shown in **Figure 1A**, the incidence of age-adjusted CCA was 3.3 per 100,000 persons in 2000, and increased to 8.8 per 100,000 persons in 2016; for comparison, the annual age-adjusted incidence of all malignancies is also depicted.

In addition, we divided all CCA cases into different subgroups based on the age and race of the patient as well as grade and stage of the tumor. First, the age-specific incidence was calculated for three age groups: <60 years, 60–69 years, and >70 years. As shown in **Figure 1B**, the incidence of CCA increased dramatically from 2000 to 2016 in patients aged <60 years, with nearly a 3-fold rise to 4.7 per 100,000 persons; among those aged 60–69 years or >70 years, there was a more modest increase of 2.5-fold.

Among the tumor grade groups, the most dramatic rise in incidence was described in patients with grade IV CCA (from 0.1 per 100,000 persons in 2000, to 0.7 per 100,000 persons in 2016; **Figure 1C**). Among the tumor stage groups, the incidence of localized CCA increased the most relative to regional or distant (from 2 per 100,000 persons in 2000 to 6.2 per 100,000 persons in 2016; **Figure 1D**). Among the ethnic groups, the incidence of CCA increased the most in Caucasians (from 2.1 per 100,000 persons in 2000 to 8.4 per 100,000 persons in 2016; **Figure 1E**).

Overall, according to the SEER 18 data, the incidence of CCA cases diagnosed increased statistically significantly from 2000 to 2016 (annual percent change (APC): 4.6, 95% confidence interval (CI) [3.4, 5.7], *P* < 0.05).

### Patient Characteristics

To compare the demographic and clinical characteristics of CCA at the different primary tumor sites, we analyzed 104,206 CCA patients identified in the SEER database (**Table 1**). We found that the median age of these patients at diagnosis was 62 years (range: 53–71 y), and the median OS was 46 months (range: 17–91 mo). Comparing the different primary tumor sites, the lowest median age at diagnosis (56 y) was that of patients with ovarian CCA, and the highest (71 years) was for patients with bladder CCA.

**Table 1:** Descriptive demographic and clinical characteristics of patients with CCA according to primary tumor site*.

Covariate	Total	KRP	Ovary	Corpus Uteri	Lung	Cervix Uteri	Bladder	Vagina	Pancreas	Breast	POM	Prostate	Liver	P value
**n**	104206	94882	4750	2557	914	496	133	112	92	89	68	59	54	
**Age (%)**														<0.001
≤30	926 (0.9)	843 (0.9)	26 (0.5)	4 (0.2)	1 (0.1)	46 (9.3)	2 (1.5)	4 (3.6)	0 (0.0)	0 (0.0)	0 (0.0)	0 (0.0)	0 (0.0)	
31-60	46677 (44.8)	42269 (44.5)	3102 (65.3)	590 (23.1)	265 (29.0)	233 (47.0)	37 (27.8)	48 (42.9)	25 (27.2)	46 (51.7)	36 (52.9)	14 (23.7)	12 (22.2)	
≥61	56603 (54.3)	51770 (54.6)	1622 (34.1)	1963 (76.8)	648 (70.9)	217 (43.8)	94 (70.7)	60 (53.6)	67 (72.8)	43 (48.3)	32 (47.1)	45 (76.3)	42 (77.8)	
**Gender, n (%)**														<0.001
Male	59587 (57.2)	58935 (62.1)	0 (0.0)	0 (0.0)	452 (49.5)	0 (0.0)	60 (45.1)	0 (0.0)	49 (53.3)	1 (1.1)	0 (0.0)	59 (100.0)	31 (57.2)	
Female	44619 (42.8)	35947 (37.9)	4750 (100.0)	2557 (100.0)	462 (50.5)	496 (100.0)	73 (54.9)	112 (100.0)	43 (46.7)	88 (98.9)	68 (100.0)	0 (0.0)	23 (42.6)	
**Year of diagnosis, n (%)**														<0.001
2000–2007	34615 (33.2)	30439 (32.1)	2043 (43.0)	1017 (39.8)	543 (59.4)	228 (46.0)	59 (44.4)	57 (50.9)	46 (50.0)	57 (50.9)	37 (54.4)	50 (84.7)	39 (72.2)	
2008–2016	69591 (66.8)	64443 (67.9)	2707 (57.0)	1540 (60.2)	371 (40.6)	268 (54.0)	74 (55.6)	55 (49.1)	46 (50.0)	32 (49.1)	31 (45.6)	9 (15.3)	15 (27.8)	
**Marital (%)**														<0.001
Married	64284 (61.7)	59559 (62.8)	2557 (53.8)	1160 (45.4)	490 (53.6)	208 (41.9)	49 (36.8)	55 (49.1)	55 (59.8)	42 (47.2)	37 (54.4)	41 (69.5)	31 (57.4)	
Widowed/Divorced	20294 (19.5)	17936 (18.9)	877 (18.5)	913 (35.7)	270 (29.5)	127 (25.6)	49 (36.8)	31 (27.7)	25 (27.2)	25 (28.1)	20 (29.4)	7 (11.9)	14 (25.9)	
Single	14864 (14.3)	13041 (13.7)	1126 (23.7)	348 (13.6)	126 (13.8)	136 (27.4)	19 (14.3)	18 (16.1)	10 (10.9)	17 (19.1)	8 (11.8)	7 (11.9)	8 (14.8)	
Unknown	4764 (4.6)	4346 (4.6)	190 (4.0)	136 (5.3)	28 (3.1)	25 (5.0)	16 (12.0)	8 (7.1)	2 (2.2)	5 (5.6)	3 (4.4)	4 (6.8)	1 (1.9)	
**Race, n(%)**														<0.001
White	88231 (84.7)	80971 (85.3)	3709 (78.1)	1902 (74.4)	785 (85.9)	396 (79.8)	108 (81.2)	76 (67.9)	81 (88.0)	69 (77.5)	53 (77.9)	47 (79.7)	34 (63.0)	
Black	7693 (7.4)	6869 (7.2)	194 (4.1)	410 (16.0)	88 (9.6)	51 (10.3)	21 (15.8)	21 (18.8)	6 (6.5)	9 (10.1)	6 (8.8)	10 (16.9)	8 (14.8)	
Other	7633 (7.3)	6427 (6.8)	832 (17.5)	232 (9.1)	39 (4.3)	48 (9.7)	4 (3.0)	14 (12.5)	5 (5.4)	10 (11.2)	9 (13.2)	1 (1.7)	12 (22.2)	
Unknown	649 (0.6)	615 (0.6)	15 (0.3)	13 (0.5)	2 (0.2)	1 (0.2)	0 (0.0)	1 (0.9)	0 (0.0)	1 (1.1)	0 (0.0)	1 (1.7)	0 (0.0)	
**Grading, n (%)**														<0.001
I	10823 (10.4)	10666 (11.2)	53 (1.1)	42 (1.6)	33 (3.6)	13 (2.6)	0 (0.0)	1 (0.9)	4 (4.3)	1 (1.1)	1 (1.5)	2 (3.4)	7 (13.0)	
II	43452 (41.7)	42604 (44.9)	397 (8.4)	149 (5.8)	164 (17.9)	47 (9.5)	7 (5.3)	8 (7.1)	14(15.2)	34 (38.2)	2 (2.9)	18 (30.5)	8 (14.8)	
III	24787 (23.8)	21250 (22.4)	1613 (34.0)	1199 (46.9)	349 (38.2)	191 (38.5)	36 (27.1)	26 (23.2)	19 (20.7)	40 (44.9)	23 (33.8)	32 (54.2)	9 (16.7)	
IV	6457 (6.2)	5019 (5.3)	806 (17.0)	456 (17.8)	31 (3.4)	79 (15.9)	40 (30.1)	13 (11.6)	0 (0.0)	2 (2.2)	9 (13.2)	2 (3.4)	0 (0.0)	
Unknown	18687 (17.9)	15343 (16.2)	1881 (39.6)	711 (27.8)	337 (36.9)	166 (33.5)	50 (37.6)	64 (57.1)	55 (59.8)	12 (13.5)	33 (48.5)	5 (8.5)	30 (55.6)	
**Stage, n (%)**														0.001
Local	71561 (68.7)	68150 (71.8)	1655 (34.8)	1075 (42.0)	273 (29.9)	210 (42.3)	75 (56.4)	0 (0.0)	5 (5.4)	59 (66.3)	0 (0.0)	36 (61.0)	23 (42.6)	
Regional	19183 (18.4)	15925 (16.8)	1756 (37.0)	925 (36.2)	284 (31.1)	196 (39.5)	24 (18.0)	0 (0.0)	30 (32.6)	23 (25.8)	0 (0.0)	10 (16.9)	10 (18.5)	
Distant	12257 (11.8)	9990 (10.5)	1288 (27.1)	457 (17.9)	339 (37.1)	78 (15.7)	23 (17.3)	0 (0.0)	53 (57.6)	6 (6.7)	0 (0.0)	11 (18.6)	12 (22.2)	
Unknown	1205 (1.2)	817 (0.9)	51 (1.1)	100 (3.9)	18 (2.0)	12 (2.4)	11 (8.3)	112 (100.0)	4 (4.3)	1 (1.1)	68 (100.0)	2 (3.4)	9 (16.7)	

*Reported as n (%), unless indicated otherwise.

The primary tumor site in these patients was significantly associated with the age at diagnosis (*P* < 0.001; **Table 1**). Patients aged ≥61 years were more likely to develop primary CCA of the KRP, corpus and cervix uteri, lungs, bladder, pancreas, prostate, or liver; while patients aged 31-60 years were more likely to have CCA of the POM. Moreover, the tumor grade was significantly different between the various primary tumor sites (*P* < 0.001; **Table 1**): patients with grade II tumors were more likely to have CCA of the KRP; while patients with grade III tumors were more likely to have CCA of the corpus and cervix uteri, lungs, breast, or prostate.

**Figure 2:**
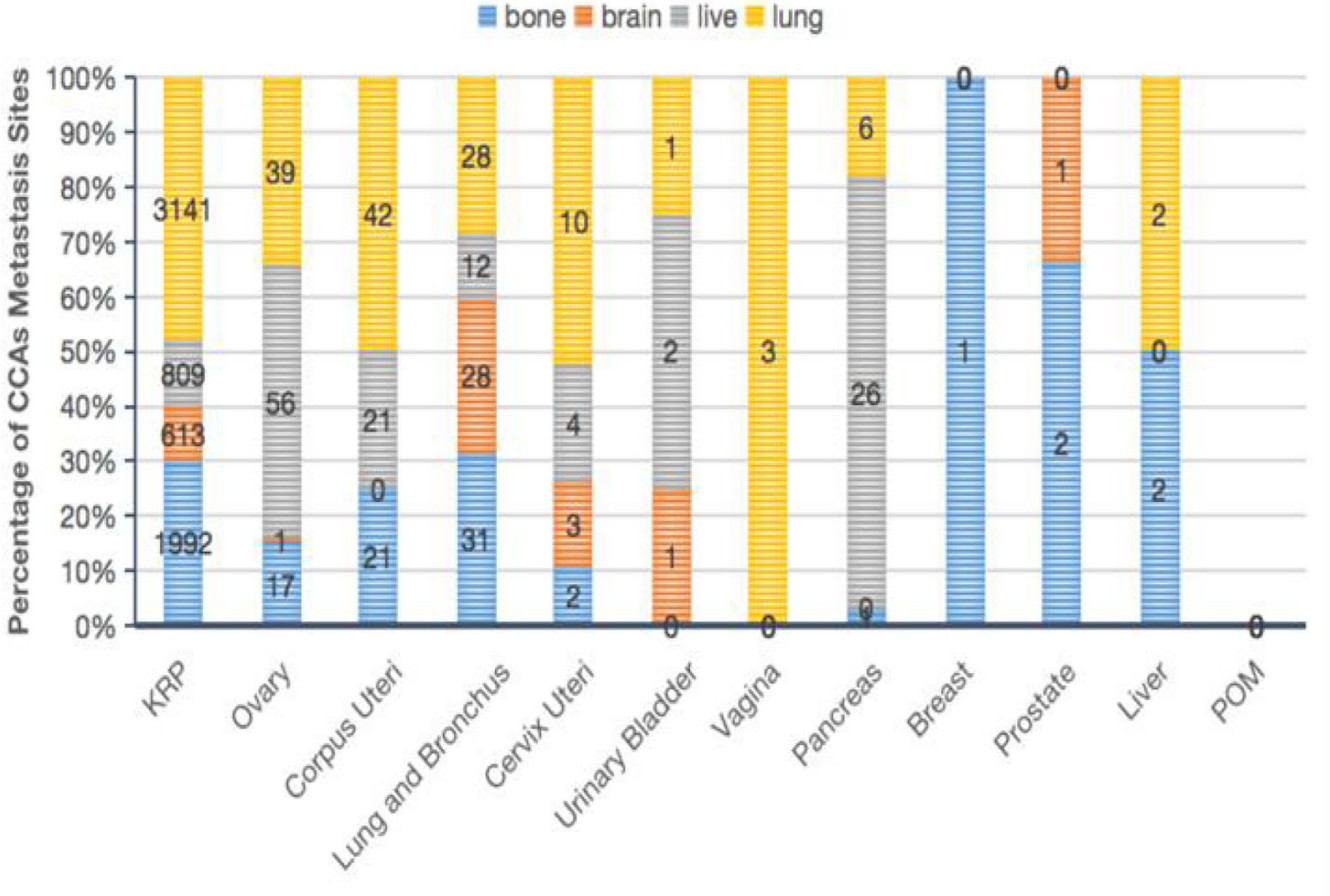
Characteristics of CCA patients according to the primary tumor site. Proportion of metastatic sites in the bone, brain, liver, or lung (B).

The tumor stage was significantly different between the various primary tumor sites (*P* = 0.001; **Table 1**): localized CCA was more likely to be in the KRP, ovary, cervix uteri, breast, prostate, or liver; while distant CCA was more likely to be in the lungs or pancreas. In addition, we determined that the metastatic sites differed depending on the type of CCA. Patients with CCA of the KRP, cervix uteri, or vagina were most likely to have lung metastases; patients with CCA of the ovary, bladder, or pancreas were more prone to have liver metastases; and patients with CCA of the lung, breast, or prostate were tended to have bone metastases (**Figure 2**).

### Survival

**Figure 3:**
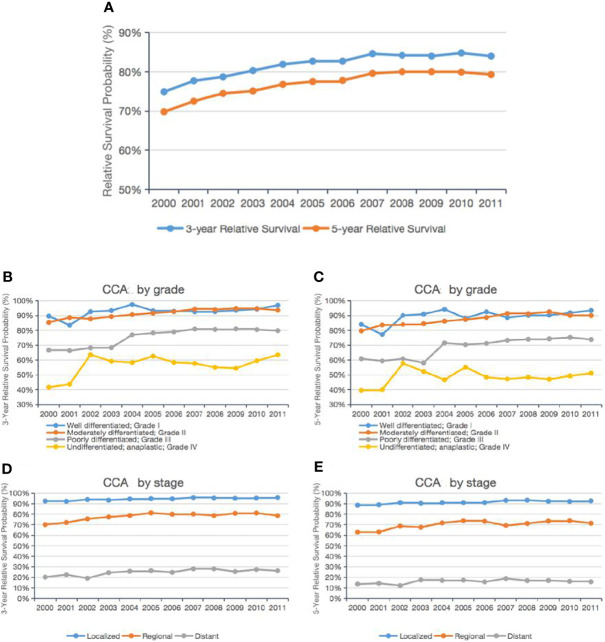
Trends in the 3-year and 5-year relative survival probabilities of CCA patients from 2000 to 2011. Trends in the 3-year and 5-year relative survival probabilities of all CCA patients **(A)**. Trends in the 3-year and 5-year relative survival probabilities of CCA patients by grade **(B, C)** and stage **(D, E)**.

We identified the latest trends in the survival of all CCA cases from 2000 to 2011 in the SEER database, relative to the general population. As shown in **Figure 3A**, the age-standardized 3-year and 5-year relative survivals increased from 2000 to 2011, rising by 9.1% and 9.5%, respectively. Specifically, when we examined the CCA cases by grade (**Figures 3B, C**), we found that the age-standardized 3-year and 5-year relative survivals of patients with grade I CCA increased from 89.6% and 84.0% in 2000 to 98.1% and 96.0% in 2011, respectively. Meanwhile, the age-standardized 3-year and 5-year relative survivals of patients with grade II CCA slightly improved from 85.4% and 79.7% in 2000 to 93.6% and 90.0% in 2011, respectively.

Those with grade III–IV CCA showed an even greater improvement: the 3-year and 5-year relative survivals of grade III patients increased from 66.7% and 41.6% in 2000 to 79.8% and 63.5% in 2011, respectively. The 3-year and 5-year relative survivals of the grade IV patients increased from 60.9% and 39.6% in 2000 to 73.8% and 51.1% in 2011. When we examined the age-standardized 3-year and 5-year relative survivals by tumor stage, we found that the relative survival of patients with localized, regional, or distant tumors had improved slightly over time (**Figures 3D, E**).

**Figure 4:**
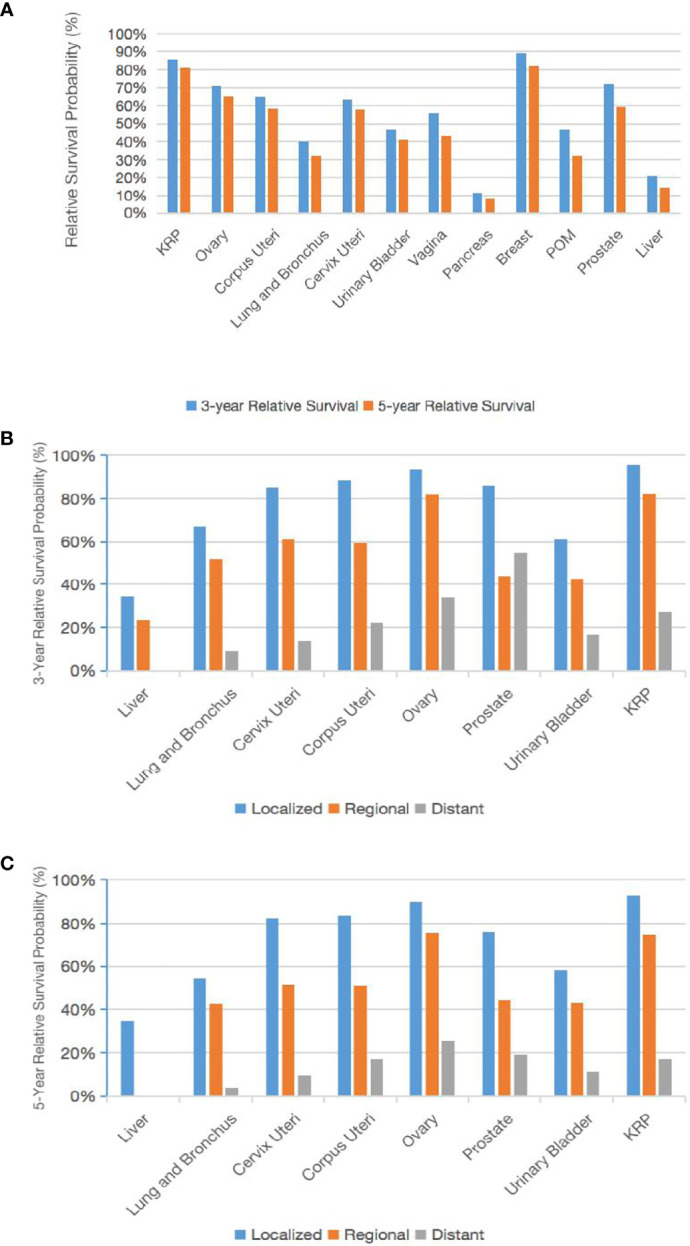
Trends in the 3-year and 5-year relative survival probabilities according to the primary tumor site. Trends in the 3-year and 5-year relative survival probabilities of patients with CCA at various primary tumor sites **(A)**. Trends in the 3-year and 5-year relative survival probabilities by stage **(B, C)**.

The age-standardized 3-year and 5-year survivals of the CCA patients relative to the general population and according to the primary tumor site were analyzed (**Figure 4A**). The largest change in 3-year to 5-year survival was for CCA of the POM (46.8% to 32.2%) or prostate (72.3% to 59.2%), and the smallest change was for CCA of the pancreas (11.4% to 8.3%) or KRP (85.6% to 81.1%). We examined the known 3-year and 5-year relative survivals of CCA of different primary tumor sites according to the tumor stage (**Figures 4B, C**). The best 5-year relative survival for regional and distant tumors was for patients with ovarian CCA.

We performed a multivariate analysis and calculated the hazard ratio for OS (**Table 2**). Age, gender, year of diagnosis, marital status, ethnicity, grade, stage, and primary tumor site were all significantly associated with OS. We found that women (HR, 0.85; 95% CI, 0.83–0.87) had a better OS than men, and patients with grade III (HR, 1.21; 95% CI, 1.16–1.26) or grade IV (HR, 1.68; 95% CI, 1.59–1.77) CCA had a worse OS than did those with grade I CCA. However, the OS was not statistically different between grade II and grade I CCA. After adjusting for other variables, regional CCA (HR, 1.91; 95% CI, 1.86–1.97) and distant CCA had a shorter median survival time compared with localized and regional CCA (14mo VS 193,103 mo, respectively). Compared with CCA of the KRP, patients with CCA of the liver had the worst OS (HR, 4.89; 95% CI, 3.71–6.44), those with CCA of the pancreas had the second worst OS (HR, 3.70; 95% CI, 2.99–4.59), and those with CCA of the ovary had the best OS (HR, 0.75; 95% CI, 0.71–0.79).

**Table 2:** Multivariate survival analysis of patients with CCA receiving diagnoses from 2000 to 2016.

Covariate	Overall Survival		3-Year Survival		5-Year Survival	
	HR	P value	HR	P value	HR	P value
**Age,y**		<0.001		<0.001		<0.001
≤30	1.00 (ref.)		1.00 (ref.)		1.00 (ref.)	
31-60	2.76 (2.16-3.52)	<0.001	1.66 (1.23-2.23)	0.001	1.94 (1.48-2.55)	<0.001
≥61	5.63 (4.41-7.19)	<0.001	2.34 (1.74-3.15)	<0.001	2.86 (2.18-3.75)	<0.001
**Gender**						
Male	1.00 (ref.)		1.00 (ref.)		1.00 (ref.)	
Female	0.85 (0.83-0.87)	<0.001	0.94 (0.91-0.97)	<0.001	0.90 (0.88-0.93)	<0.001
**Year of diagnosis**						
2000–2007	1.00 (ref.)		1.00 (ref.)		1.00 (ref.)	
2008–2016	0.87 (0.85-0.89)	<0.001	0.42 (0.41-0.44)	<0.001	0.37 (0.36-0.38)	<0.001
**Marital**		<0.001		<0.001		<0.001
Married	1.00 (ref.)		1.00 (ref.)		1.00 (ref.)	
Widowed/Divorced	1.44 (1.40-1.48)	<0.001	1.28 (1.23-1.32)	<0.001	1.28 (1.25-1.33)	<0.001
Single	1.26 (1.22-1.30)	<0.001	1.15 (1.10-1.20)	<0.001	1.18 (1.14-1.23)	<0.001
Unknown	0.99 (0.93-1.05)	0.619	0.97 (0.90-1.05)	0.483	0.93 (0.87-0.99)	0.032
**Race**		<0.001		<0.001		<0.001
White	1.00 (ref.)		1.00 (ref.)		1.00 (ref.)	
Black	1.20 (1.15-1.25)	<0.001	1.20 (1.14-1.26)	<0.001	1.18 (1.13-1.24)	<0.001
Other	0.91 (0.87-0.95)	<0.001	1.00 (0.94-1.06)	0.909	0.96 (0.92-1.02)	0.163
Unknown	0.26 (0.18-0.36)	<0.001	0.27 (0.18-0.42)	<0.001	0.31 (0.22-0.46)	<0.001
**Grading**		<0.001		<0.001		<0.001
I	1.00 (ref.)		1.00 (ref.)		1.00 (ref.)	
II	0.96 (0.92-1.00)	0.059	0.84 (0.79-0.90)	<0.001	0.88 (0.83-0.93)	<0.001
III	1.21 (1.16-1.26)	<0.001	1.04 (0.97-1.11)	0.317	1.08 (1.02-1.24)	0.012
IV	1.68 (1.59-1.77)	<0.001	1.37 (1.27-1.48)	<0.001	1.48 (1.39-1.58)	<0.001
Unknown	1.48 (1.41-1.55)	<0.001	1.35 (1.26-1.44)	<0.001	1.41 (1.33-1.49)	<0.001
**Stage**		<0.001		<0.001		<0.001
Local	1.00 (ref.)		1.00 (ref.)		1.00 (ref.)	
Regional	1.91 (1.86-1.97)	<0.001	1.64 (1.57-1.71)	<0.001	1.66 (1.60-1.72)	<0.001
Distant	8.29 (8.06-8.53)	<0.001	3.92 (3.78-4.07)	<0.001	4.49 (4.34-4.64)	<0.001
Unknown	2.90 (2.66-3.16)	<0.001	2.56 (2.30-2.83)	<0.001	2.33 (2.12-2.56)	<0.001
**Site**		<0.001				
KRP	1.00 (ref.)		1.00 (ref.)		1.00 (ref.)	
Ovary	0.75 (0.71-0.79)	<0.001	0.87 (0.82-0.93)	<0.001	0.90 (0.85-0.96)	0.001
Corpus Uteri	1.16 (1.09-1.23)	<0.001	1.10 (1.02-1.18)	0.01	1.22 (1.25-1.31)	<0.001
Lung	2.10 (1.95-2.26)	<0.001	1.55 (1.42-1.70)	<0.001	1.78 (1.64-1.93)	<0.001
Cervix Uteri	1.29 (1.13-1.48)	<0.001	1.10 (0.94-1.28)	0.229	1.46 (1.26-1.68)	<0.001
Bladder	1.64 (1.32-2.03)	<0.001	1.34 (1.06-1.69)	0.016	1.69 (1.35-2.12)	<0.001
Vagina	1.04 (0.80-1.35)	0.762	0.83 (0.61-1.12)	0.227	0.96 (0.73-1.26)	0.762
Pancreas	3.70 (2.99-4.59)	<0.001	2.77 (2.22-3.45)	<0.001	3.56 (2.86-4.43)	<0.001
Breast	1.00 (0.71-2.41)	0.992	1.14 (0.65-2.01)	0.65	1.16 (0.76-1.78)	0.493
POM	1.77 (1.33-2.36)	<0.001	0.96 (0.68-1.35)	0.804	0.90 (0.67-1.22)	0.502
Prostate	0.94 (0.68-1.30)	0.728	0.89 (0.55-1.43)	0.624	0.72 (0.48-1.07)	0.102
Liver	4.89 (3.71-6.44)	<0.001	2.12 (158-2.86)	<0.001	2.46 (1.84-3.28)	<0.001

We analyzed the latest trends of the OS during 2000–2007 and 2008–2016. Compared with 2000–2007, the risk of death in CCA diagnosed in 2008–2016 was less by 13% (HR, 0.87; 95% CI, 0.85–0.89). We calculated the 3-year and 5-year hazard ratios through multivariate analysis, and the patients with grade II CCA had better 3-year (HR, 0.84; 95% CI, 0.79–0.90) and 5-year (HR, 0.88; 95% CI, 0.83–0.93) survivals than did those patients with grade I CCA. All of the above comparisons are significant (*P* < 0.001).

**Figure 5:**
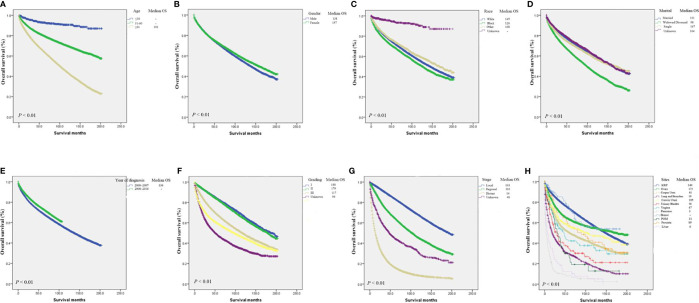
Kaplan-Meier analysis of survival by age **(A)**, gender **(B)**, race **(C)**, marital status **(D)**, year of diagnosis **(E)**, grade **(F)**, stage **(G)**, primary tumor site **(H)**.

Furthermore, the Kaplan-Meier curve and median OS were analyze for age, gender, year of diagnosis, marital status, race, grade, stage, and primary tumor site. The results were consistent with multivariate cox regression analysis. Specifically, the population with Widowed/Divorced had the worst prognosis, with the median OS of only 98 months (**Figure 5D**). CCA patients with distant-stage had a median OS of only 14 months, compared with 193 and 103 months for localized and regional, respectively. The median OS was best for breast cancer CCA (not achieving median OS) and ovarian cancer CCA (175 mo) (**Figure 5G**), while the median OS was worst for pancreas (3 mo) and liver (6 months). All of these differences in OS were significant (P < 0.001; **Figures 5A–H**).

## Discussion

In this study, we used the SEER database to report the largest number of CCA cases for the first time, focusing on incidence, demographic characteristics, and prognostic factors. We found that the age-adjusted incidence of CCA increased from 3.3 per 100,000 persons in 2000 to 8.8 per 100,000 persons in 2016, which is a 2.7-fold increase. This increase may be due in part to factors such as increased early diagnosis of these tumors and insurance coverage (15–17). The survival of CCA patients also increased significantly over time, reflecting that comprehensive treatment based on surgery has improved for CCA patients in recent years (18–20).

Although the incidence increased across all ages, grades, stages, and races during this period, the incidence increased the most for grade IV CCA, then localized stage; and American Indians and Alaskan Natives among races. However, it is unclear whether these differences are due to underlying dietary habits, environmental factors, biological factors, or health care models. Furthermore, CCA is associated with variables such as older age, white, male, grade II and local stage. CCA also occurs most frequently in the kidneys and ovaries. Patients with kidney or ovarian CCA are more likely to have metastasis to the lungs and liver compared with other solid CCAs, respectively, a finding consistent with previous reports (21, 22).

In addition, we analyzed the relative survival of patients with all grades and stages of CCA and found that survival over time increased the most in patients with grade IV CCA. One possible explanation is that surgery-based comprehensive treatment models for high-grade CCA have improved in recent years (23, 24). The results of the multivariate survival analysis showed that age, gender, year of diagnosis, marital status, ethnicity, disease stage, grade, and primary tumor site are important predictors of the OS of CCA patients. We also found that the most useful predictor of prognosis in patients with CCA is probably the primary tumor site. Therefore, the results of our research above can be used as a practical guide for clinicians.

Previous studies have shown that CCA is formed by the abnormal accumulation of glycogen. The prognosis is poor for patients with CCA of the kidney, uterus, ovary, bladder, or breast (11, 13, 25, 26). The present research showed that compared with CCA of the kidney, CCA of the liver and pancreas has a relatively worse OS, while CCA of the ovary has a better prognosis.

The poor prognosis of CCA patients may be due to several biomolecular mechanisms. Glycogen metabolism has recently been recognized as an important pathway for metabolic reprogramming in cancer cells. Others have reported that tumorigenesis and progression inhibit hypoglycemic glycogen metabolism and thereby inhibit active oxygen levels and p53-dependent cell senescence (14). Furthermore, tumor cells can mobilize glycogen to promote glycolysis and increase cancer cell proliferation, invasion, and metastasis through various signaling pathways such as p38α mitogen-activated protein kinase and mammalian target of rapamycin (27, 28).

Abnormal glycogen accumulation can serve as an important energy supply that compensate for nutritional deficiencies in the tumor microenvironment (2). Therefore, targeting glucose metabolism is considered an important approach for cancer treatment (4, 8). However, there is currently no effective treatment for CCA. A deep understanding of cancer glycogen metabolism is needed to identify novel targeted treatments for these glycogen-rich cancers, to serve as options to surgery for comprehensive treatment.

There are some limitations to our research. First, this study was retrospective and had an inherent selection bias. Second, our study did not include factors such as the quality of surgery and systemic treatments, which may confound the results. Finally, the SEER database does not capture a number of possible prognostic indicators such as insurance status, eating habits, and environmental factors, which may also influence treatment decisions and survival outcomes.

## Conclusions

In this large-scale study, we evaluated the incidence of CCA as well as the demographics and survival of CCA patients. Over time, the incidence of CCA and patient survival increased. In particular, the highest increases were reported for grade IV CCA compared with all subgroups, which may be due to increased diagnosis of the disease and improved treatment. Our research will help clinicians fully understand the natural history and progression of these glycogen-rich tumors as well as provide a theoretical basis for identifying novel targeted therapies.

## Data Availability Statement

The original contributions presented in the study are included in the article/**Supplementary Material**. Further inquiries can be directed to the corresponding authors.

## Author Contributions

Conceptualization, YG and XY. Data curation, AS and XD. Formal analysis, NM, AZ, and XD. Funding acquisition, XY. Investigation, SM. Methodology, RW and AZ. Project administration, YG and XY. Resources, WM. Software, YG. Supervision, CL. Validation, JL and WZ. Visualization, ZZ and WZ. Writing – review & editing, YG and XY. All authors have read and approved the manuscript.

## Funding

This work was supported in part by grants from the Shanghai Science Committee Foundation (#19411967700).

## Conflict of Interest

The authors declare that the research was conducted in the absence of any commercial or financial relationships that could be construed as a potential conflict of interest.

## Publisher’s Note

All claims expressed in this article are solely those of the authors and do not necessarily represent those of their affiliated organizations, or those of the publisher, the editors and the reviewers. Any product that may be evaluated in this article, or claim that may be made by its manufacturer, is not guaranteed or endorsed by the publisher.

## Abbreviations

CCA, clear cell adenocarcinoma; HR, hazard ratio; KRP, kidney and renal pelvis; OS, overall survival; POM, peritoneum, omentum, and mesentery; SEER, Surveillance, Epidemiological, and End Results.
